# Effects of Intraovarian Platelet-Rich Plasma on Ovarian Reserve Markers, Oocyte Development, and Pregnancy Outcomes in Women with Diminished Ovarian Reserve: An Uncontrolled Retrospective Before–After Study

**DOI:** 10.3390/jcm15145674

**Published:** 2026-07-20

**Authors:** Hüseyin Kayaalp, Hakan Çoksuer, Sertaç Ayçiçek, Abdurrahman Açıkgöz, Seyhmus Tunc, Kevser Arkan, Hüseyin Altas, Serap Mutlu Özcelik Otcu

**Affiliations:** 1 Perinatology Unit, Department of Obstetrics and Gynecology, University of Health Sciences, Ankara Bilkent City Hospital, Ankara 06800, Turkey; 2 Department of Obstetrics and Gynecology, IVF Clinic, Private Diyarlife Hospital, Diyarbakır 21300, Turkey; coksuer@gmail.com; 3 Department of Obstetrics and Gynecology, Diyarbakir Gazi Yasargil Research and Training Hospital, Diyarbakir 21070, Turkey; doctorsertac@gmail.com (S.A.); drseyhmustunc@hotmail.com (S.T.); 4 Department of Radiology, Private Diyarlife Hospital, Diyarbakır 21300, Turkey; Abdurrahman57@hotmail.com; 5 Department of Obstetrics and Gynecology, Division of Gynecologic Oncology, Diyarbakir Gazi Yasargil Research and Training Hospital, Diyarbakir 21070, Turkey; kevser.toprak1989@gmail.com; 6 Department of Obstetrics and Gynecology, Division of Gynecologic Oncology, University of Health Sciences, Ankara Bilkent City Hospital, Ankara 06800, Turkey; huso.altas@gmail.com; 7 Infertility Unit, Department of Obstetrics and Gynecology, Diyarbakir Gazi Yasargil Research and Training Hospital, Diyarbakir 21070, Turkey; serozcelik@hotmail.com

**Keywords:** platelet-rich plasma, diminished ovarian reserve, infertility, antral follicle count, 2PN zygote, pregnancy outcomes

## Abstract

**Background**: The aim of this study was to investigate the impact of intraovarian platelet-rich plasma (PRP) treatment on ovarian reserve markers, hormonal profiles, oocyte yield, fertilization potential, and pregnancy outcomes in infertile women with diminished ovarian reserve. **Methods**: This retrospective single-center study was conducted at a tertiary referral center between January 2021 and December 2024 in the Department of Obstetrics and Gynecology, IVF Clinic, Private Diyarlife Hospital, Diyarbakır, Turkey. A total of 78 infertile women with diminished ovarian reserve (DOR) were included. DOR was defined in the present study as an AMH level < 1.1 ng/mL and/or an antral follicle count (AFC) < 5 follicles. Pre-PRP and post-PRP measurements were compared in the same patients. Hormonal and clinical parameters, including prolactin, thyroid-stimulating hormone (TSH), follicle-stimulating hormone (FSH), anti-Müllerian hormone (AMH), estradiol, endometrial thickness, gonadotropin dosage, antral follicle count (AFC), mature metaphase II (MII) oocyte count, and two-pronuclear (2PN) zygote count, were evaluated before and after PRP treatment. **Results**: Significant increases were observed in AMH and prolactin levels, whereas FSH levels significantly decreased following PRP treatment (all *p* < 0.001). Post-PRP AFC, MII oocyte count, and 2PN zygote count were significantly higher than pre-PRP values (all *p* < 0.001). A total of 21 pregnancies (26.9%) were achieved, including 17 clinical pregnancies (81.0% of pregnancies; 21.8% of all patients), 4 biochemical pregnancies (19.0% of pregnancies; 5.1% of all patients), 11 miscarriages (52.4% of pregnancies; 14.1% of all patients), and 6 live births (28.6% of pregnancies; 7.7% of all patients). Significant positive correlations were identified between post-PRP AFC and MII oocyte count (r = 0.803, *p* < 0.001), post-PRP AFC and 2PN zygote count (r = 0.624, *p* < 0.001), and MII oocyte count and 2PN zygote count (r = 0.728, *p* < 0.001). **Conclusions**: Intraovarian PRP administration was associated with improvements in ovarian reserve markers, oocyte maturation, and fertilization-related outcomes in women with DOR. Significant increases in AFC, MII oocyte count, and 2PN zygote count were observed after PRP treatment. However, these improvements in ovarian reserve markers were not associated with significant differences between women who achieved pregnancy and those who did not. Because of the retrospective design and the absence of a control group, these findings should be interpreted as associations rather than evidence of a causal treatment effect. Although clinical pregnancies and live births were observed during follow-up, larger, multicenter randomized controlled trials are required to confirm these findings and to further evaluate the effect of PRP on reproductive outcomes.

## 1. Introduction

One of the most common problems encountered in female infertility is diminished ovarian reserve (DOR), which significantly reduces the chance of pregnancy by limiting the response to assisted reproductive techniques [1,2]. Particularly in women aged 35 years and older, the decline in antral follicle count and the decrease in anti-Müllerian hormone (AMH) levels are among the critical parameters determining treatment success [3]. In recent years, intraovarian platelet-rich plasma (PRP) administration has emerged as an experimental biological approach aimed at re-stimulating ovarian function, offering promising results.

PRP is a plasma derivative obtained from the patient’s own blood that contains a high concentration of growth factors. Through mechanisms such as tissue regeneration, angiogenesis, and cell proliferation, PRP has the potential to exert regenerative effects within the ovarian stroma [4,5]. Clinical studies have reported a decrease in follicle-stimulating hormone (FSH) levels, along with significant increases in AMH levels and antral follicle counts following PRP injection [6,7]. Furthermore, some patients have been observed to resume spontaneous ovulation after PRP treatment, and improved oocyte yield has been reported during in vitro fertilization (IVF) cycles [8].

Observational and prospective studies suggest that PRP may positively influence not only biochemical parameters but also fertility outcomes such as embryo development, fertilization rate, and blastocyst quality [9,10]. However, evidence from randomized controlled trials remains limited, and these studies have not yet demonstrated consistent improvements in clinically important reproductive outcomes [8]. Therefore, further well-designed randomized controlled trials are required to better establish the efficacy of PRP. In this context, our study aims to evaluate the biological and clinical effects of PRP on ovarian reserve.

## 2. Materials and Methods

### 2.1. Study Population

This study was designed as a retrospective before–after (pre–post) single-center study and was conducted between January 2021 and December 2024 in the Department of Obstetrics and Gynecology, IVF Clinic, Private Diyarlife Hospital, Diyarbakır, Turkey. A total of 78 infertile women aged 18–45 years who underwent intraovarian PRP injection were included. Although no universally accepted diagnostic criteria exist for diminished ovarian reserve (DOR), reduced anti-Müllerian hormone (AMH) and antral follicle count (AFC) are widely accepted markers of decreased ovarian reserve. In the present study, DOR was defined as an AMH level < 1.1 ng/mL and an AFC < 5 follicles. These thresholds are consistent with the abnormal ovarian reserve test component of the ESHRE Bologna criteria and with the low-prognosis groups (POSEIDON Groups 3 and 4), both of which incorporate reduced ovarian reserve markers [1,3]. All 78 included patients fulfilled these predefined DOR criteria, with an AMH level < 1.1 ng/mL and an AFC < 5 follicles.

The inclusion criteria were: at least 12 months of infertility despite regular unprotected intercourse and DOR, defined in the present study as an AMH level < 1.1 ng/mL and an AFC < 5 follicles; normal karyotype analysis; normal uterine anatomy confirmed by transvaginal ultrasonography (TVUS) or hysterosalpingography (HSG); and regular menses or oligomenorrhea. Exclusion criteria were: history of chemotherapy, radiotherapy, or pelvic surgery affecting ovarian function; stage III–IV endometriosis; polycystic ovary syndrome (PCOS) or autoimmune disease; active pelvic infection or contraindications for transvaginal procedures; serious systemic diseases such as uncontrolled diabetes, hypertension, or cardiovascular disease; and patients who refused PRP or were noncompliant with follow-up. Clinical, demographic, and obstetric characteristics of the participants were recorded. Pre-PRP and post-PRP measurements of the same patients were compared. The following data were collected before and after PRP: AFC, prolactin, thyroid-stimulating hormone (TSH), FSH, AMH, estradiol, endometrial thickness, gonadotropin dosage, mature metaphase II (MII) oocyte count, two-pronuclear (2PN) zygote count, and post-PRP pregnancy outcomes (clinical pregnancies, biochemical pregnancies, ectopic pregnancies, miscarriages, ongoing pregnancies, and live births). This study was conducted retrospectively using existing medical records. Written informed consent was routinely obtained from all patients before the PRP procedure as part of standard clinical practice. Ethical approval for the retrospective review of patient records and analysis of the patient data was obtained from the Ethics Committee of Gazi Yaşargil Training and Research Hospital in accordance with the Declaration of Helsinki (approval dated 11 July 2025; No. 564).

### 2.2. PRP Preparation and Administration

For autologous PRP preparation, approximately 20 mL of peripheral venous blood was collected from each patient under sterile conditions into citrate-containing tubes. A standardized two-step centrifugation protocol was used to obtain the platelet-rich plasma fraction [5,7]. The autologous PRP was prepared using the T-LAB PRP kit (T-LAB, Bursa, Turkey) and processed with a T-LAB S-106 NXT (T-LAB, Bursa, Turkey) centrifuge according to the manufacturer’s instructions. The first centrifugation was performed at 1200 rpm (204× *g*) for 10 min to separate erythrocytes from the plasma fraction, followed by a second centrifugation at 2500 rpm (886× *g*) for 10 min to concentrate platelets. This procedure yielded approximately 2 mL of PRP for administration.

PRP injection was performed during the early follicular phase of the menstrual cycle (days 2–4) under transvaginal ultrasound guidance [6,7]. A total of approximately 2 mL of PRP was administered using a 17-gauge aspiration needle under transvaginal ultrasound guidance, with approximately 1 mL injected into each ovary. No procedure-related adverse events or complications were observed.

### 2.3. Evaluation and Follow-Up

The primary outcome measures were AMH, FSH, prolactin, basal serum estradiol, and TSH levels; AFC; MII oocyte count; and 2PN zygote count, all assessed before PRP and within the first 3 months after the procedure. All included patients had undergone an IVF/ICSI stimulation cycle before PRP treatment. The pre-PRP gonadotropin dosage, MII oocyte count, 2PN zygote count, endometrial thickness, and trigger-day serum estradiol levels were obtained from each patient’s last IVF/ICSI cycle before PRP treatment, whereas the corresponding post-PRP values were obtained from the first IVF/ICSI cycle performed after PRP treatment. Basal serum estradiol levels were measured during the early follicular phase (cycle days 2–3), whereas trigger-day serum estradiol levels were measured on the day of final oocyte maturation during controlled ovarian stimulation. Thus, all pre- and post-treatment outcomes were compared within the same patient by comparing paired pre- and post-PRP IVF/ICSI cycles. Controlled ovarian stimulation protocols were individualized according to patient characteristics and the treating physician’s clinical judgment. Ovarian stimulation was generally initiated with 300 IU recombinant follicle-stimulating hormone (Gonal-F^®^, Merck KGaA, Darmstadt, Germany), administered alone or in combination with human menopausal gonadotropin (Menopur^®^, Ferring Pharmaceuticals, Kiel, Germany), as clinically indicated. A GnRH antagonist (Cetrotide^®^, Merck KGaA, Darmstadt, Germany) was administered when clinically appropriate. Final oocyte maturation was triggered using recombinant human chorionic gonadotropin (Ovitrelle^®^, Merck KGaA, Darmstadt, Germany). All patients underwent fresh embryo transfer following ICSI during the first post-PRP IVF/ICSI cycle. All reported pregnancies resulted from fresh embryo transfer during this first post-PRP IVF/ICSI cycle; no spontaneous pregnancies occurred. Hormone levels were analyzed using validated chemiluminescent immunoassay kits. Antral follicle count was assessed by the same specialist using transvaginal ultrasonography to minimize interobserver variability. Biochemical pregnancy was defined as a positive serum β-hCG test without ultrasonographic evidence of an intrauterine gestational sac. Clinical pregnancy was defined as the presence of an intrauterine gestational sac with fetal cardiac activity on transvaginal ultrasonography. Miscarriage was defined as spontaneous pregnancy loss. Live birth was defined as the delivery of a live infant. Ongoing pregnancy was defined as a viable intrauterine pregnancy. Pregnant patients were followed until pregnancy outcome (miscarriage or live birth), whereas reproductive outcomes for non-pregnant patients were evaluated during the first IVF/ICSI cycle after PRP treatment. Complete pre- and post-PRP clinical, hormonal, and embryological data were available for all 78 included patients; therefore, no patient was excluded from the analyses because of missing data.

### 2.4. Statistical Analysis

Data analyses were performed using SPSS version 25.0 (SPSS Inc., Chicago, IL, USA). The normality of data distribution was evaluated using the Kolmogorov–Smirnov and Shapiro–Wilk tests. Continuous variables were expressed as median (interquartile range, IQR), whereas categorical variables were presented as frequencies and percentages. Comparisons between pre-PRP and post-PRP measurements were performed using the Wilcoxon signed-rank test. Effect sizes (r) were calculated for all paired comparisons to quantify the magnitude of the observed differences. Comparisons between pregnant and non-pregnant patients were performed using the Mann–Whitney U test. Correlations between variables were assessed using Spearman’s rank correlation coefficient. A *p*-value < 0.05 was considered statistically significant.

## 3. Results

A total of 78 patients were included in the study. The patients’ age, BMI, and duration of infertility are presented in Table 1.

Comparison of hormonal parameters before and after PRP treatment is shown in Table 2. Significant increases were observed in AMH and prolactin levels, whereas FSH levels significantly decreased following PRP treatment (all *p* < 0.001). No significant differences were found in basal serum estradiol (E2) or TSH levels between the pre-PRP and post-PRP periods (*p* > 0.05) (Table 2).

As shown in Table 3, post-PRP AFC, MII oocyte count, and 2PN zygote count were significantly higher than pre-PRP values (all *p* < 0.001).

In addition, endometrial thickness and trigger-day serum estradiol levels increased significantly after PRP treatment, whereas gonadotropin dosage significantly decreased (all *p* < 0.001) (Table 4).

A total of 21 pregnancies (26.9% of all patients) were achieved following IVF/ICSI treatment after intraovarian PRP. Among these pregnancies, 17 (81.0%) were clinical pregnancies, and 4 (19.0%) were biochemical pregnancies. Overall, 11 patients (14.1% of the study cohort; 52.4% of all pregnancies) experienced miscarriage, whereas 6 patients (7.7% of the study cohort; 28.6% of all pregnancies) achieved live birth. No ectopic or ongoing pregnancies were observed. Pregnancy outcomes after intraovarian PRP treatment are presented in Table 5.

Table 6 compares clinical and reproductive parameters between pregnant and non-pregnant patients. No statistically significant differences were observed between the groups regarding age, BMI, duration of infertility, post-PRP AMH, post-PRP AFC, MII oocyte count, or 2PN zygote count (all *p* > 0.05).

Spearman correlation analysis demonstrated strong positive correlations between post-PRP AFC and MII oocyte count (r = 0.803, *p* < 0.001), post-PRP AFC and 2PN zygote count (r = 0.624, *p* < 0.001), and MII oocyte count and 2PN zygote count (r = 0.728, *p* < 0.001). No significant correlations were identified between 2PN zygote count and age, BMI, duration of infertility, or post-PRP AMH levels (all *p* > 0.05) (Table 7).

## 4. Discussion

This study aimed to investigate the effects of PRP administration on ovarian reserve markers, hormonal parameters, oocyte development, and reproductive outcomes in infertile women with diminished ovarian reserve. Our findings demonstrated a significant increase in AMH levels, a decrease in FSH levels, and notable increases in AFC, MII oocyte count, and 2PN zygote count following PRP treatment. Experimental and preclinical studies have hypothesized that PRP may enhance follicular activity and exert regenerative effects within the ovarian stroma. However, these proposed mechanisms have not yet been confirmed in clinical studies and they cannot be inferred from the findings of the present study [11,12,13,14].

The median age of the 78 patients included in the study was 40 years, with a median BMI of 31 kg/m^2^ and a median duration of infertility of 9 years. These findings indicate that PRP is primarily applied in women of advanced reproductive age with long-standing infertility and reduced ovarian reserve. Consistent with our findings, previous studies have also predominantly evaluated PRP treatment in similar patient populations [12,13].

After PRP treatment, AMH levels increased significantly, whereas FSH levels decreased and prolactin levels increased (*p* < 0.001). In contrast, no significant changes were observed in basal serum estradiol or TSH levels. In addition, significant increases were observed in AFC, MII oocyte count, and 2PN zygote count following PRP treatment. Previous experimental and preclinical studies have hypothesized that PRP may enhance follicular activity and exert regenerative effects within the ovarian stroma. However, these proposed mechanisms cannot be inferred from the findings of the present study and should instead be regarded as hypotheses requiring further confirmation in clinical studies [15,16,17]. Although prolactin levels increased significantly after PRP treatment, the biological significance of this finding remains uncertain. Therefore, the observed increase should be interpreted with caution and warrants further investigation in prospective studies.

A significant increase was observed in AFC, MII oocyte count, and 2PN zygote count following PRP treatment (*p* < 0.001). These observations are consistent with improvements in follicular, oocyte-related, and fertilization-related parameters after PRP treatment. However, because of the uncontrolled retrospective study design, these findings should be interpreted as temporal associations rather than evidence that PRP directly improved these outcomes. Similar improvements in ovarian reserve markers and reproductive outcomes have been reported in previous studies. Cakiroglu et al. demonstrated significant increases in AFC, AMH levels, mature oocyte yield, and fertilization outcomes following intraovarian PRP administration. Likewise, Najafian et al. reported improvements in ovarian reserve markers, oocyte development, and pregnancy outcomes after PRP treatment. Furthermore, Farimani et al. documented increased oocyte yield and successful live births following intraovarian PRP injection in women with POR [7,18,19].

In our study, trigger-day serum estradiol levels and endometrial thickness were higher after PRP treatment, whereas gonadotropin dosage was lower. Similar observations have been reported in previous studies. However, because of the uncontrolled retrospective design of the present study, these findings should be interpreted as temporal associations rather than evidence that PRP directly improved ovarian response or endometrial development. Collectively, our findings add to the growing body of observational evidence regarding intraovarian PRP treatment in women with DOR; however, they do not establish clinical efficacy and should be confirmed in adequately powered randomized controlled trials [6,7,18,20].

A total of 21 pregnancies (26.9%) were achieved following IVF/ICSI treatment after intraovarian PRP, including 17 clinical pregnancies, 4 biochemical pregnancies, 11 miscarriages, and 6 live births. Although PRP treatment was associated with encouraging pregnancy outcomes, the miscarriage rate exceeded the live birth rate in our cohort. Therefore, despite the observed improvements in ovarian reserve markers and embryological outcomes, these findings should be interpreted with caution, and the present study cannot conclude that PRP improves live birth rates. Similar findings have been reported in previous studies. Tülek and Kahraman reported that PRP treatment may improve oocyte and embryo quality in women with poor ovarian response, although its impact on clinical pregnancy and live birth rates remains uncertain. Likewise, Petryk et al. documented successful pregnancies and live births following PRP administration in women with diminished ovarian reserve. Furthermore, Farimani et al. reported live births after intraovarian PRP injection, supporting the potential reproductive benefits of PRP in poor ovarian responders [19,21,22].

Previous studies have suggested that improvements in oocyte yield and oocyte maturation following PRP treatment may be associated with higher pregnancy rates. Safarova et al. reported that the increase in retrieved oocytes and MII oocytes count was more pronounced among women who achieved pregnancy after PRP treatment [23]. In contrast, in our study, no significant differences were observed between pregnant and non-pregnant women regarding post-PRP AFC, MII oocyte count, 2PN zygote count, or AMH levels. These findings suggest that although PRP treatment may improve ovarian reserve markers and oocyte-related parameters, pregnancy success cannot be explained solely by these factors and may also be influenced by multiple additional variables, including embryo quality, endometrial receptivity, sperm quality, and patient-specific characteristics. Therefore, improvements in surrogate markers should not be interpreted as evidence of improved reproductive success or live birth outcomes.

Correlation analysis revealed strong positive associations between post-PRP AFC and MII oocyte count, post-PRP AFC and 2PN zygote count, and MII oocyte count and 2PN zygote count. These findings indicate that women with higher post-PRP AFC also tended to have higher MII oocyte and 2PN zygote counts. Similar associations between ovarian reserve markers, oocyte development, and reproductive outcomes have been reported in previous studies [7,18,20]. However, these correlations should be interpreted with caution, as they are biologically expected. A greater antral follicle count naturally yields more mature oocytes and, consequently, a higher number of fertilized embryos. Therefore, these associations likely reflect the intrinsic biological relationship between follicular development, oocyte yield, and embryo development rather than an independent effect of PRP.

### 4.1. Limitations

The main limitations of this study include its retrospective single-center design, the relatively small sample size, and the absence of a control group. Because of the retrospective study design, an a priori sample size calculation was not performed. Instead, all eligible patients who met the inclusion criteria during the study period were included in the analysis. In addition, the retrospective nature of the study precludes establishing a causal relationship between PRP treatment and the observed outcomes. Furthermore, the relatively small number of pregnancies and live births limits the interpretation of reproductive outcomes. The absence of a parallel control group for pregnancy outcomes also limits conclusions regarding the direct effect of PRP on reproductive success. Moreover, the observed improvements may partly be explained by regression to the mean, selection bias, differences in individualized ovarian stimulation protocols, or differences in measurement timing rather than a direct effect of PRP. Additionally, certain technical parameters of the PRP product, including platelet concentration, leukocyte content, and platelet activation status, were not routinely documented in the medical records and therefore could not be reported retrospectively. Because this was an exploratory retrospective study, no formal adjustment for multiple comparisons was performed. Therefore, the observed improvements in ovarian reserve markers and pregnancy outcomes should be interpreted with caution. Larger prospective randomized controlled studies are needed to confirm these findings and to determine the long-term effects of PRP treatment on reproductive outcomes. Accordingly, the observed changes cannot be distinguished from the potential effects of regression to the mean, natural biological variation, or other time-dependent factors.

### 4.2. Strengths

Despite these limitations, this study provides a comprehensive evaluation of hormonal parameters, ovarian reserve markers, oocyte-related outcomes, and pregnancy outcomes following intraovarian PRP treatment. In addition, the availability of paired pre- and post-treatment measurements enabled within-patient comparisons, thereby reducing interindividual variability. Furthermore, all embryological outcomes and gonadotropin dosage were compared using paired pre- and post-PRP IVF/ICSI cycles within the same patients.

## 5. Conclusions

In conclusion, intraovarian PRP administration was associated with improvements in ovarian reserve markers, oocyte development, and fertilization-related outcomes in infertile women with DOR. Compared with pre-treatment measurements, higher AMH levels, lower FSH levels, and higher AFC, MII oocyte counts, and 2PN zygote counts were observed after treatment. Clinical pregnancies and live births were observed during follow-up. These improvements in ovarian reserve markers were not associated with significant differences between women who achieved pregnancy and those who did not. However, because of the retrospective design and the absence of a control group, these findings should be interpreted as associations observed within an uncontrolled cohort rather than as evidence of a causal treatment effect. Nevertheless, larger prospective randomized controlled trials with standardized PRP protocols and live birth as the primary outcome are required to confirm these findings and clarify the effect of PRP on reproductive outcomes.

## Figures and Tables

**Table 1:** Clinicodemographic characteristics of women undergoing intraovarian PRP treatment.

	*n*	Median	Minimum	Maximum	Interquartile Range (IQR)
Age (years)	78	40.0	21.0	45.0	12.25
BMI (kg/m^2^)	78	31.0	21.0	44.0	10.0
Duration of infertility (years)	78	9.0	5.0	24.0	9.5

Descriptive statistics are presented as median, minimum, maximum, and interquartile range (IQR).

**Table 2:** Comparison of hormonal parameters before and after intraovarian PRP treatment.

Parameter	Pre-PRP Median (IQR)	Post-PRP Median (IQR)	*p*-Value	Effect Size (r)
AMH (ng/mL)	0.04 (0.06)	0.38 (0.32)	<0.001	0.869
FSH (mIU/mL)	25.8 (16.8)	16.0 (8.0)	<0.001	0.879
Prolactin (ng/mL)	12.0 (6.0)	19.0 (13.0)	<0.001	0.559
Basal serum estradiol (pg/mL)	15.6 (26.0)	19.5 (9.5)	0.944	0.007
TSH (mIU/mL)	1.9 (1.0)	1.8 (1.5)	0.987	0.017

AMH: anti-Müllerian hormone. FSH: Follicle-Stimulating Hormone. TSH: Thyroid-Stimulating Hormone. Statistical analysis: Wilcoxon signed-rank test. Effect sizes (r) are reported for paired comparisons. Data are presented as median (IQR). A *p*-value < 0.05 was considered statistically significant.

**Table 3:** Comparison of ovarian reserve and oocyte-related parameters before and after PRP.

Parameter	Pre-PRP Median (IQR)	Post-PRP Median (IQR)	*p*-Value	Effect Size (r)
AFC	0.0 (0.3)	2.0 (2.0)	<0.001	0.885
MII oocyte count	0.0 (0.0)	1.0 (1.0)	<0.001	0.892
2PN zygote count	0.0 (0.0)	1.0 (0.0)	<0.001	0.926

AFC: Antral follicle count. MII oocyte count: Mature metaphase II oocyte count. 2PN zygote count: Two-pronuclear (2PN) zygote count. Statistical analysis was performed using the Wilcoxon signed-rank test. Effect sizes (r) are reported for paired comparisons. Data are presented as median (IQR). A *p*-value < 0.05 was considered statistically significant.

**Table 4:** Comparison of endometrial thickness, estradiol levels, and gonadotropin dosage before and after PRP.

Parameter	Pre-PRP Median (IQR)	Post-PRP Median (IQR)	*p*-Value	Effect Size (r)
Endometrial thickness (mm)	9.0 (1.0)	10.0 (2.0)	<0.001	0.885
Trigger-day serum estradiol (pg/mL)	230.0 (120.0)	313.0 (119.0)	<0.001	
Gonadotropin dosage (IU)	4500.0 (750.0)	4275.0 (750.0)	<0.001	0.925

Statistical analysis: Wilcoxon signed-rank test. Effect sizes (r) are reported for paired comparisons. Data are presented as median (IQR). A *p*-value < 0.05 was considered statistically significant.

**Table 5:** Pregnancy outcomes after PRP.

	Patient-Based (*n* = 78)	Pregnancy-Based (*n* = 21)
Total pregnancies (IVF/ICSI), *n* (%)	21 (26.9)	-
Clinical pregnancies, *n* (%)	17 (21.8)	17 (81.0)
Biochemical pregnancies, *n* (%)	4 (5.1)	4 (19.0)
Miscarriages, *n* (%)	11 (14.1)	11 (52.4)
Live births, *n* (%)	6 (7.7)	6 (28.6)
Ectopic pregnancies, *n* (%)	0 (0.0)	0.0 (0.0)
Ongoing pregnancies, *n* (%)	0 (0.0)	0.0 (0.0)

Values are presented as number and percentage (*n* (%)). Patient-based percentages were calculated using all included patients (*n* = 78), whereas pregnancy-based percentages were calculated using the total number of pregnancies (*n* = 21).

**Table 6:** Comparison of clinical and reproductive parameters between pregnant and non-pregnant patients after PRP treatment.

Parameters	Pregnant (*n* = 21)	Non-Pregnant (*n* = 57)	*p*-Value
Age (years)	39.0 (8.0)	41.0 (12.0)	0.074
BMI (kg/m^2^)	31.0 (12.0)	31.0 (10.0)	0.861
Duration of infertility (years)	9.0 (8.0)	9.0 (12.0)	0.527
Post-PRP AMH (ng/mL)	0.42 (0.26)	0.35 (0.32)	0.865
Post-PRP AFC	2.0 (1.0)	2.0 (1.0)	0.150
MII oocyte count	2.0 (1.0)	1.0 (1.0)	0.664
2PN zygote count	1.0 (0.0)	1.0 (0.0)	0.775

AFC: Antral follicle count. MII oocyte count: mature metaphase II oocyte. 2PN zygote count: Two-pronuclear (2PN) zygote count. AMH: Anti-Müllerian hormone. Data are presented as median (IQR). Comparisons were performed using the Mann–Whitney U test. A *p*-value < 0.05 was considered statistically significant.

**Table 7:** Spearman correlation analysis between post-PRP ovarian reserve markers and reproductive outcomes.

Variable 1	Variable 2	Spearman’s Rho (r)	*p*-Value
Age (years)	2PN zygote count	−0.097	0.400
BMI (kg/m^2^)	2PN zygote count	−0.052	0.650
Duration of infertility (years)	2PN zygote count	−0.096	0.403
Post-PRP AMH (ng/mL)	2PN zygote count	−0.059	0.606
Post-PRP AFC	MII oocyte count	0.803	<0.001
Post-PRP AFC	2PN zygote count	0.624	<0.001
MII oocyte count	2PN zygote count	0.728	<0.001

AFC: Antral follicle count. MII oocyte count: Mature metaphase II oocyte count. 2PN zygote count: Two-pronuclear (2PN) zygote count. AMH: Anti-Müllerian hormone. Correlation coefficients were calculated using Spearman’s rank correlation test. A *p*-value < 0.05 was considered statistically significant.

## Data Availability

The data presented in this study are available on request from the corresponding author. The data are not publicly available due to privacy and ethical restrictions.

## Abbreviations

AFC	Antral Follicle Count
AMH	Anti-Müllerian Hormone
BMI	Body Mass Index
E2	Estradiol
DOR	Diminished Ovarian Reserve
ICSI	Intracytoplasmic Sperm Injection
ESHRE	European Society of Human Reproduction and Embryology
FSH	Follicle-Stimulating Hormone
HSG	Hysterosalpingography
IQR	Interquartile Range
IVF	In Vitro Fertilization
MII	Mature Metaphase II Oocyte
2PN	Two-pronuclear zygote
PRP	Platelet-Rich Plasma
TSH	Thyroid-Stimulating Hormone
TVUS	Transvaginal Ultrasonography
